# *In vivo* characterization of cerebrovascular impairment induced by amyloid β peptide overload in glymphatic clearance system using swept-source optical coherence tomography

**DOI:** 10.1117/1.NPh.10.1.015005

**Published:** 2023-02-16

**Authors:** Yao Yu, Ning Zhang, Ben Xiang, Ning Ding, Jian Liu, Jiangmei Huang, Min Zhao, Yuqian Zhao, Yi Wang, Zhenhe Ma

**Affiliations:** a Northeastern University at Qinhuangdao, School of Control Engineering, Qinhuangdao, China; b Hebei Key Laboratory of Micro-Nano Precision Optical Sensing and Measurement Technology, Qinhuangdao, China; c Northeastern University, College of Information Science and Engineering, Shenyang, China; d First Hospital of Qinhuangdao, Department of Pathology, Qinhuangdao, China

**Keywords:** amyloid *β*, Alzheimer’s disease, glymphatic system, cerebrovasculature, optical coherence tomography

## Abstract

**Significance:**

Antiamyloid β (Aβ) immunotherapy is a promising therapeutic strategy for Alzheimer’s disease (AD) but generates large amounts of soluble Aβ peptides that could overwhelm the clearance pathway, leading to serious side effects. Direct implications of Aβ in glymphatic drainage transport for cerebral vasculature and tissue are not well known. Studies are needed to resolve this issue and pave the way to better monitoring abnormal vascular events that may occur in Aβ-modifying therapies for AD.

**Aim:**

The objective is to characterize the modification of cerebral vasculature and tissue induced by soluble Aβ abundantly present in the glymphatic clearance system.

**Approach:**

$A\beta_{1-42}$ peptide was injected intracerebroventricularly and swept-source optical coherence tomography (SS-OCT) was used to monitor the progression of changes in the brain microvascular network and tissue *in vivo* over 14 days. Parameters reflecting vascular morphology and structure as well as tissue status were quantified and compared before treatment.

**Results:**

Vascular perfusion density, vessel length, and branch density decreased sharply and persistently following peptide administration. In comparison, vascular average diameter and vascular tortuosity were moderately increased at the late stage of monitoring. Endpoint density gradually increased, and the global optical attenuation coefficient value decreased significantly over time.

**Conclusions:**

Aβ burden in the glymphatic system directly contributes to cerebrovascular structural and morphological abnormalities and global brain tissue damage, suggesting severe deleterious properties of soluble cerebrospinal fluid-Aβ. We also show that OCT can be used as an effective tool to monitor cerebrovascular dynamics and tissue property changes in response to therapeutic treatments in drug discovery research.

## 1 Introduction

Amyloid β (Aβ) peptides are naturally occurring cleavage products of the amyloid precursor protein via sequential proteolytic processes called β- and γ-secretase.^1^ A primary neuropathological hallmark of Alzheimer’s disease (AD) involves extracellular Aβ deposition to form senile plaques, leading to neuronal death and cognitive degeneration.^2^ Therefore, Aβ deposit elimination has been a major focus of Aβ-based AD therapeutics over the past decades. Currently, there are more than one hundred agents in clinical trials for AD therapeutics.^3^ Although, Aducanumab, an anti-Aβ human monoclonal antibody, was approved by the U.S. Food and Drug Administration in 2021 for early AD.^4^ The efficacy of this drug remains highly controversial.^5^^,^^6^ In addition, amyloid-related imaging abnormalities (ARIA) often co-occur during anti-Aβ immunotherapy (also seen with Aducanumab).^7^^,^^8^ The high incidence of ARIA makes it a major adverse effect of Aβ-targeted immunotherapies, which hinders the drug to reach the primary clinical endpoints.^9^^–^^11^ ARIA can be detected by magnetic resonance imaging (MRI) as hyperintensities on fluid-attenuated inversion recovery but is mostly asymptomatic.^7^^,^^12^ It is suggested that the phenomena might be due to the breakdown of the Aβ plaque, liberating abundant amount of soluble Aβ peptides that overwhelm the capacity of Aβ clearance through perivascular cerebrospinal fluid (CSF) bulk flow system.^13^^–^^17^ This leads to greater Aβ deposition in the vessel wall and causes a self-reinforcing cycle, thereby exacerbating the development of cerebral amyloid angiopathy (CAA), a common feature of AD that has been reported to affect 80% to 90% of AD cases.^9^^,^^13^^,^^14^^,^^18^^,^^19^ Besides, under pathological conditions of mild cognitive impairment and AD, the balance between Aβ production and clearance is disrupted so the decline of the clearance system, rather than the Aβ overproduction, contributes to Aβ deposition.^20^^,^^21^ Perivascular drainage is a major route of amyloid clearance from brain^22^ and a key contributor is the glymphatic system, which involves CSF entering the brain along paravascular spaces surrounding penetrating arteries and exchanging with interstitial fluid, which then exit the brain alongside veins.^23^^–^^25^ It has been reported that ventricular CSF clearance of Aβ is negatively related with the magnitude of brain Aβ deposition.^26^ Consequently, efficient Aβ clearance via the glymphatic transport system is crucial for maintaining brain health and efficacious AD therapeutics; therefore, it is necessary to monitor the clearance dysfunction. Although ARIA occurs during anti-Aβ treatment, it does not correlate with the speed of Aβ elimination and might not have adequate sensitivity.^27^ Pathological modification to the cerebral vascular architecture represents one of the earliest events in the AD pathological cascade and are evident earlier than the classical hallmark of the disease, Aβ deposition, and the onset of cognitive impairment.^28^^–^^30^ While the significance of vascular dysfunction is widely appreciated, its pathological changes caused by the Aβ clearance deficit through glymphatic transport are not clear. This raises the urgent need to characterize and quantitively evaluate vascular modifications after Aβ overload in CSF in preclinical settings. This may help us unravel the direct vascular consequences of Aβ clearance dysfunction and may also guide better treatment adjustments in patients receiving Aβ-targeted immunotherapy.

*In vivo* small animal brain imaging has facilitated rapid advances in neuroscience. Many imaging modalities have been applied to visualize cerebral vasculature in animal models to study AD-related pathology. Confocal microscopy has high resolution (<1  μm) but very limited imaging depth (<500  μm) and imaging area (not large enough for an entire mouse brain). Positron emission tomography (PET) and MRI can image the whole-brain vasculature, but their spatial resolution is relatively low, with PET>1  mm and MRI>100  μm.^31^ Besides, they both have low temporal resolution. Comparatively, the penetration depth of optical coherence tomography (OCT) is enough to visualize mouse neocortex (≤1  mm) where the Aβ deposit is widespread and is one of the most and earliest affected areas in AD progression and ARIA.^9^ OCT also has good imaging resolution (∼μm) and range [one scan can cover the entire mouse brain with swept-source OCT (SS-OCT)^32^]. SS-OCT possesses a balance of penetration depth, resolution, and imaging range, ideal for monitoring cerebral vascular morphology and tissue properties throughout the mouse brain. Other advantages include noninvasiveness, no need for contrast agent, and fast data acquisition.^33^ OCT angiography (OCTA) is a functional extension of OCT that provides a three-dimensional (3D) map of blood perfusion in tissue down to the capillary level.^34^ OCTA has been successfully used to image microvascular networks in a variety of tissues *in vivo*, such as brain,^32^ retina,^35^ skin,^36^ skeletal muscles,^37^ and microvascular anastomosis.^38^ It is widely used as a diagnostic tool in ophthalmology,^35^ dermatology,^39^ and cardiovascular.^40^ In addition to vascular imaging, analysis of optical attenuation coefficient (OAC) from OCT data enables monitoring and quantitative assessment of brain tissue damage and edema.^41^^–^^43^

In this study, we utilized SS-OCT to investigate the effect of CSF-Aβ peptide on cortical microvascular network and tissue for a period of 2 weeks. Parameters such as vascular perfusion density (VPD), vessel length (VL), vascular average diameters (VAD), vessel tortuosity (VT), branch density, and endpoint density were evaluated to demonstrate comprehensive modification of cerebrovascular morphology. In addition, we have also shown the overall deterioration of brain tissue over time by means of OAC. We believe this paper will advance our current understanding of the cerebrovascular and cortical tissue changes in the context of dysfunctional Aβ-clearance system, which may potentially serve as biomarkers for better treatment response and facilitate the development of effective AD drugs.

## 2 Methods

### 2.1 Animal Preparation

All procedures were approved by the Institutional Animal Care Committee of Northeastern University. All efforts were made to minimize animal suffering and to reduce the number of animals used. All mice were housed in a certified facility, with a constant temperature (22°C) under a 12/12-h light/dark cycle and had access to food and water *ad libitum*. C57Bl/6NCr male mice between 8 and 12 weeks of age were used in all experiments. Mice were anesthetized using sodium pentobarbital (3%, 5  mg/100  g, IP). Heart rate of the animal was monitored and kept at 500 beats per minute, and the temperature was monitored using a rectal probe and regulated with a heating pad. The anesthetized mouse was fixed on a stereotaxic apparatus (ST-5ND-C) with ear bars and a clamping device. After the fur was shaved, the skin was cut along the midline of the skull to expose the interparietal bone. $A\beta_{1-42}$ (A9810; Sigma-Aldrich, St. Louis, Missouri) was dissolved in 10% DMSO/PBS and incubated overnight at 37°C before use. The peptide (1  μg/μl) was injected intracerebroventricularly into the lateral ventricle of the mouse at a rate of 1  μl/min for 3 min. The coordinates for stereotactic injection were 0.5 mm posterior, 1 mm lateral, and 2.5 mm ventral from the bregma, according to The Mouse Brain in Stereotaxic Coordinates (2001).

### 2.2 System and Data Acquisition

**Fig. 1:**
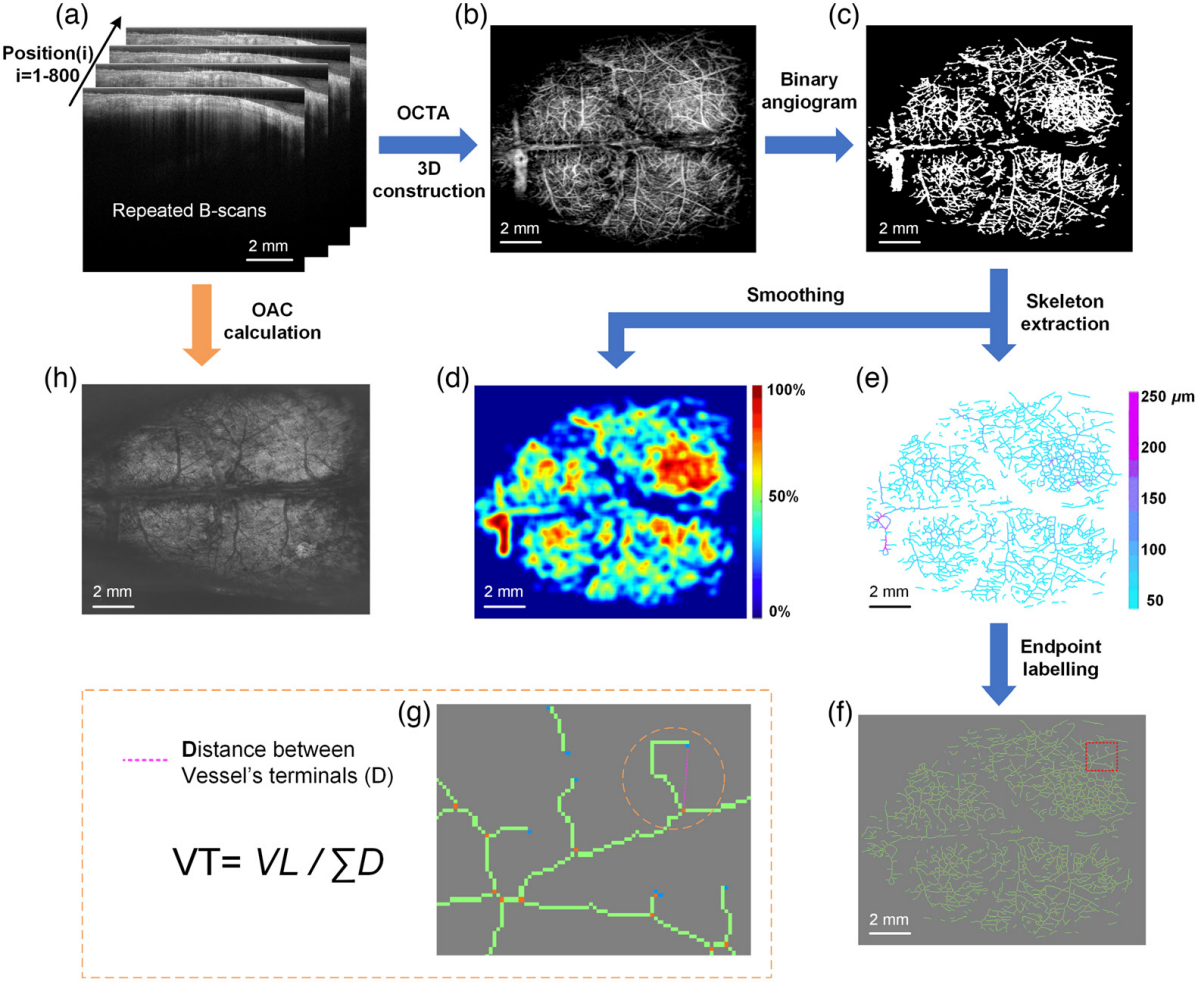
Overview of data processing procedures. (a) The original structural images acquired by SS-OCT. Scans are repeated four times at each position and a total of 800 positions were collected. (b) OCT angiogram. (c) Binary angiogram. (d) VPD image. (e) Vascular skeleton image. (f) Vascular skeleton image with vessel branch terminals labeled (orange dots: branch nodes, blue dots: endpoints). (g) Enlarged view of the red box in (f). (h) OAC image.

A wide-range SS-OCT system was used to scan the mouse brain before and after peptide injection. The light source used was a swept-source with a central wavelength of 1310 nm and a bandwidth of 100 nm. The system operates at a line scanning speed of 200 kHz and provides an axial resolution of 7.5  μm in air. An object lens with a focal length of 50 mm was adopted to achieve a lateral resolution of 16  μm. Each OCTA volume consisted of 800 slice positions (slow scan, y direction) with each position repeated four times to obtain angiography based on motion contrast [Fig. 1(a)]. In fast scan (X direction), each B-scan included 1000 A-scans with each A-scan contained 800 pixels, providing imaging depths greater than 7 mm. A set of 3D data covered the entire mouse cerebral cortex with a field of view of 12 mm (X direction) × 10 mm (Y direction).

### 2.3 Image Processing

A single data acquisition performed by SS-OCT can provide both vascular and structural information from which several parameters can be quantified (Fig. 1). Figure 1(a) shows four repeated B-scans at one position. OCTA utilizes the intrinsic motion contrast caused by dynamically moving particles (e.g., red blood cells) to distinguish functional blood vessels from static tissue. Specifically, OCTA compares the decorrelation signal (differences in the backscattered OCT signal intensity or amplitude) between sequential OCT B-scans taken at the same cross-section to construct a map of blood flow. Between repeated B-scans, the blood flow signal has a small correlation, and the static tissue has a large correlation. The decorrelation operation is to remove the static tissue (with large correlation) and retain the blood flow signals (with small correlation). Therefore, we used decorrelation values D(x,z) to extract blood flow signals. Each decorrelation frame was computed from two adjacent amplitude frames $B_{n}(x,z)$ and $B_{n+1}(x,z)$, and the average decorrelation image $\overset{¯}{D}(x,z)$ at each position is obtained as

$$\overset{¯}{D}(x,z)=1-\frac{1}{N-1}\overset{N-1}{\underset{n=1}{\sum }}\frac{B_{n}(x,z)B_{n+1}(x,z)}{\left[\frac{1}{2}B_{n}{\left(x,z\right)}^{2}+\frac{1}{2}B_{n+1}{\left(x,z\right)}^{2}\right]},$$ (1)

where x and z are the lateral and depth indices of the B-scan images, and n denotes the B-scan slice index. N is the number of repeated B-scans for each position. Thus, the vasculature was extracted, and Fig. 1(b) shows a representative OCT angiogram. Vascular information of the cerebral cortex was extracted using Amira 3D visualization software, and the final *en face* angiogram was reconstructed using maximum projection method after all positions were processed. To quantify vascular parameters, blood vessel segmentation was then performed using locally adaptive region growing algorithm.^44^ The method confines the region growing process to local regions. A distance d from the current candidate pixel to the boundary of the current local region was set. The rules are described as

$${\overset{¯}{S}}_{ij}=\overset{i+d}{\underset{x=i-d}{\sum }}\overset{j+d}{\underset{y=j-d}{\sum }}\{S_{xy}|S_{xy}\in \Omega_{l}\}/N_{l}\;,$$ (2)

$$|S_{ij}-{\overset{¯}{S}}_{ij}|<T_{ij},$$ (3)

where $\Omega_{l}$ represents the determined vessel pixels within the local region of (2d+1)×(2d+1); $N_{l}$ is the number of total pixels in $\Omega_{l}$; ${\overset{¯}{S}}_{ij}$ is the average value of all pixels in $\Omega_{l}$; $S_{ij}$ is the candidate pixel currently to be judged whether it is smaller than the threshold $T_{ij}$,^44^ and if it is less than $T_{ij}$, then it is included in the blood vessel pixels. Specifically, $T_{ij}$ represents the dynamic threshold when judging the current candidate pixel. Threshold selection is a key factor in determining the success of an algorithm. In this paper, $T_{ij}$ varies with ${\overset{¯}{S}}_{ij}$. The method used makes $T_{ij}$ proportional to ${\overset{¯}{S}}_{ij}$:

$$T_{ij}=\lambda {\overset{¯}{S}}_{ij},$$ (4)

where λ is the scaling factor. Here, the value of λ is set to 0.63 to accommodate local contrast. Depending on the growth condition, the algorithm is repeated, terminating when there are no absorbable pixels. Finally, we obtained a binary image of an OCT angiogram as shown in Fig. 1(c). This improved algorithm enables vessel identification in circumstances of weak blood flow signals and high background noise.

In the binarized blood vessel image, the blood vessel pixel value is 1, and the background pixel value is 0. Using a large size (30×30  pixels) mean filter template to perform mean filtering on the binarized blood vessel image to obtain a local blood perfusion density map [Fig. 1(d)]. Each pixel value in Fig. 1(d) represents the blood perfusion density of the local area (30×30  pixels) surrounding the point. The larger the value, the richer the local blood flow, and the smaller the value, the rarer the blood flow.

The segmented vessel images undergo two processes of distance transformation and skeleton extraction. The distance transformation is to detect the shortest distance from each “vessel pixel” to the nearest “background pixel” in the binary image. Each pixel value in the distance transformed image reflects the shortest distance from that point to the nonvascular region. Obviously, the shortest distance from the central pixel of the blood vessel to the nonvascular area is the blood vessel radius. Then, nonmaximum suppression in the cross-sectional direction is performed on the distance-transformed vessel image, and only the pixel values on the central axis of the blood vessel are retained. The blood vessel skeleton is acquired through removing the blood vessel pixel values that are not on the central axis. As a result, a vascular skeleton map [Fig. 1(e)] is obtained. In the figure, the blood vessel skeleton reflects the VL and each pixel value in the skeleton map represents the radius of the blood vessel (coded in color).

All endpoints and branch nodes are detected and identified from the vascular skeleton, starting from any one endpoint, and searching for the next endpoint/branch node along the vascular skeleton. Points that connect only one vessel branch are defined as endpoints, and points that connect three or more vessel branches are defined as branch nodes. Points connecting two vessel branches are regarded as ordinary vessel pixels. The labeled points were marked and is shown in Fig. 1(f). VT is defined as the ratio of the total VL to the sum of the straight-line distances between two endpoints or an endpoint to a branch node [shown in Fig. 1(g)]. Then, it calculates the straight-line distance (D) between these two points. In this way, the entire image is traversed to obtain the sum of straight-line distances of all blood vessels, ∑D.

The OAC calculations were performed on the image obtained through averaging four repeated B-scans. The previously proposed optimized depth-resolved estimation method^45^ was used. Each pixel in OCT dataset was converted to the corresponding OAC value using Eq. (5):

$$\mu [z]=\frac{I[z]}{2\Delta \sum_{i=z+1}^{N}I[i]+\frac{I[N]}{\mu [N]}},$$ (5)

where I[z] is the OCT signal of a pixel and μ[z] is the corresponding OAC, both at depth z. Δ is the pixel size (usually relevant to the axial resolution of the OCT system). N is pixel numbers within a certain range of depth. I[N] is the OCT signal of the last point N. To determine μ[N], the data taken from the end of the imaging depth were fitted to an exponential curve with a fitting model of y=a·exp(−2μz)+b. After all B-scan images were converted into corresponding OAC images, the OAC at a depth of 0.5 to 1 mm in the cortex was extracted from the 3D OAC data for averaging, and the OAC *en face* projection was shown in Fig. 1(h).

### 2.4 Parameter Quantification

Vascular parameters such as VPD, VAD, VL, and VT derived from the binarized OCT angiograms were quantified. VPD is defined as the ratio between “the number of blood vessel pixels” and “the total number of pixels in the angiogram” and can be calculated from the entire brain or from regional areas. To calculate VAD, the binarized OCT angiograms undergo a distance transformation process before the central axes of vessels are extracted [Fig. 1(e)]. The signal intensity reflected by the color represents the vessel radius, and VAD can be calculated by doubling the signal intensity value. VL is defined as the total length of the vascular skeleton. To calculate VT, vessel branch nodes (orange dots) and endpoints (blue dots) are marked [Figs. 1(f) and 1(g)]. VT is obtained from dividing VL by the sum of Euclidean distance (straight lengths) between terminals of a branch [Fig. 1(g) and equation].^46^^,^^47^ A straight vessel has tortuosity of 1 and the more tortuous a vessel, the larger its tortuosity value. Branch number is calculated as the total number of orange dots in the whole brain, and the branch density is the value of branch number divided by VL. Endpoint number is the total number of blue dots, and endpoint density is the ratio of endpoint number to VL.

### 2.5 Data Analysis and Statistics

Statistical analyses were conducted using GraphPad Prism (Graph-Pad Software Inc., San Diego, California). All experiments in the study were performed at six times (unless otherwise specified), and data are presented as mean ± S.E.M. Statistical differences were assessed with an unpaired two-tailed Student’s t-test when comparing two groups. Value of P<0.05 was considered statistically significant.

## 3 Results

### 3.1 Whole Brain Images Obtained by SS-OCT

**Fig. 2:**
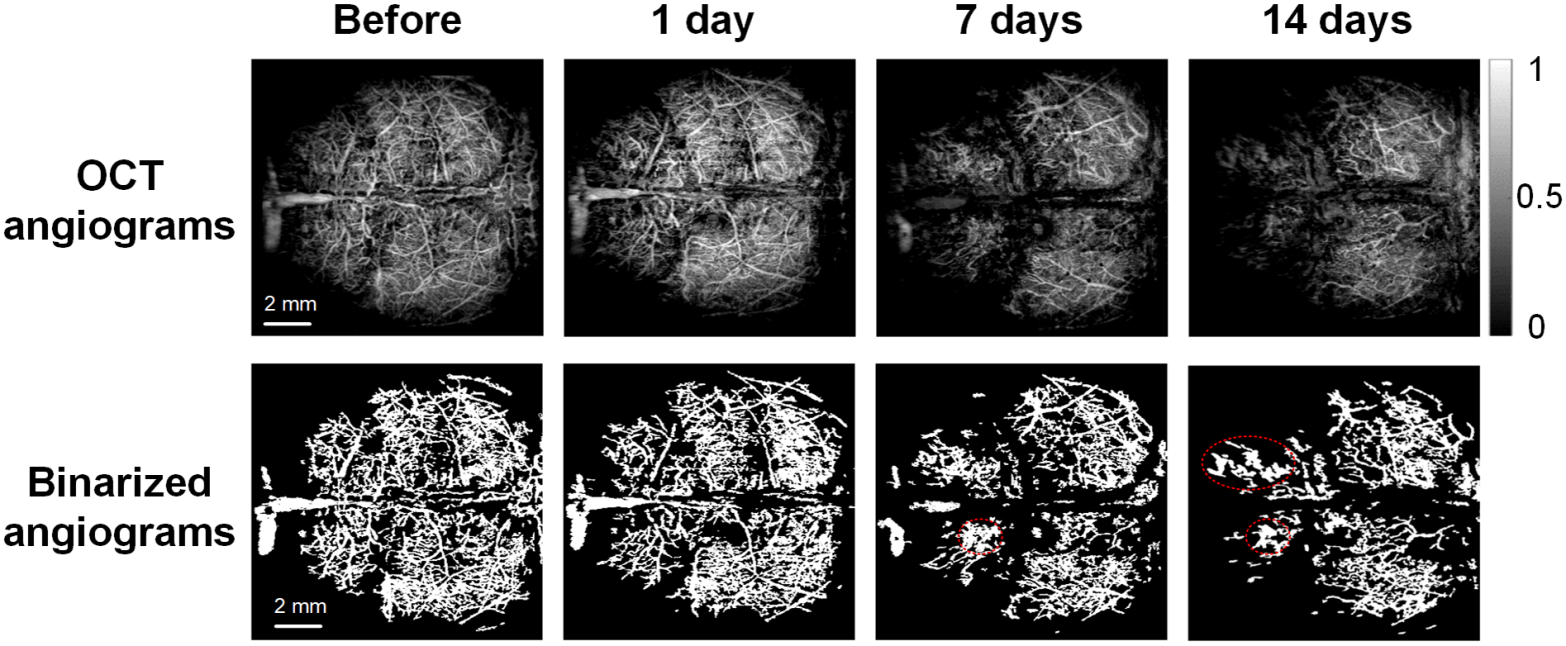
Cerebral vasculature images of mouse brain obtained using SS-OCT. The first row shows the original OCT angiograms, the second row presents the corresponding binarized angiograms at different time points. Dotted circles indicate the “vascular clusters” or “vascular bundles.”

Mice were given Aβ peptide through ventricular injection and the whole brain was monitored with SS-OCT over 2 weeks. To investigate changes in cerebral vascular morphology, OCTA images were processed to display the binarized angiographies, VPD maps, and vascular skeleton diagrams, as shown in Fig. 1. Representative brain images show that following peptide administration, capillary rarefaction was observed and became more severe with time (Fig. 2).

### 3.2 Effect of Aβ Peptide Overload in CSF on Cortical Vascular Morphology

**Fig. 3:**
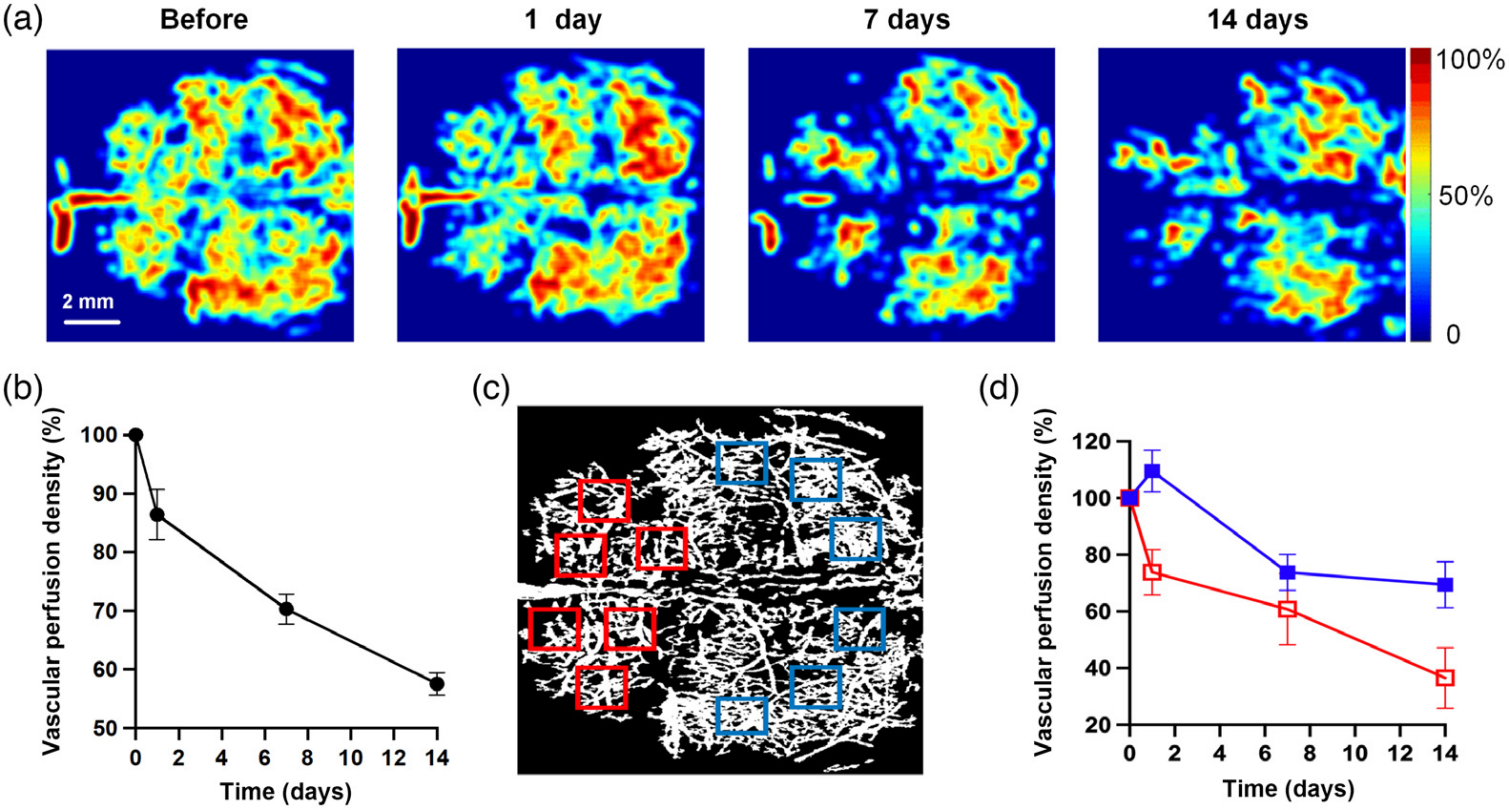
Aβ peptide induces VPD reduction in mouse brain. VPD maps derived from OCT data before and 1, 7, and 14 days after amyloid infusion in CFS (a). Data were expressed as percentage values relative to before treatment. The percentage of (b) total VPD and (d) local VPD selected in the anterior (red squares in c) and posterior (blue squares in c) parts of the brain, were also plotted over monitoring time, respectively.

Figure 3(a) shows the VPD maps of OCT angiographies of a representative mouse brain before and after 1, 7, and 14 days of amyloid peptide injection. Both the imaging results [Fig. 3(a)] and the quantification result [Fig. 3(b)] revealed that the VPD decreased drastically after Aβ peptide was applied. On days 1, 7, and 14, the VPD reduced to 86.5%, 70.3%, and 57.6% of that before treatment, respectively [Fig. 3(b)]. This may indicate insufficient vascular perfusion properties with a large amount of Aβ in the CSF pathway. To study the pattern of VPD changes closely, 1  mm×1  mm regions were arbitrarily selected in *en face* images of the whole brain [Fig. 3(c)]. It was found that the VPD values in the anterior brain section (red squares) decreased more rapidly than those in the posterior part (blue squares) [Fig. 3(d)].

**Fig. 4:**
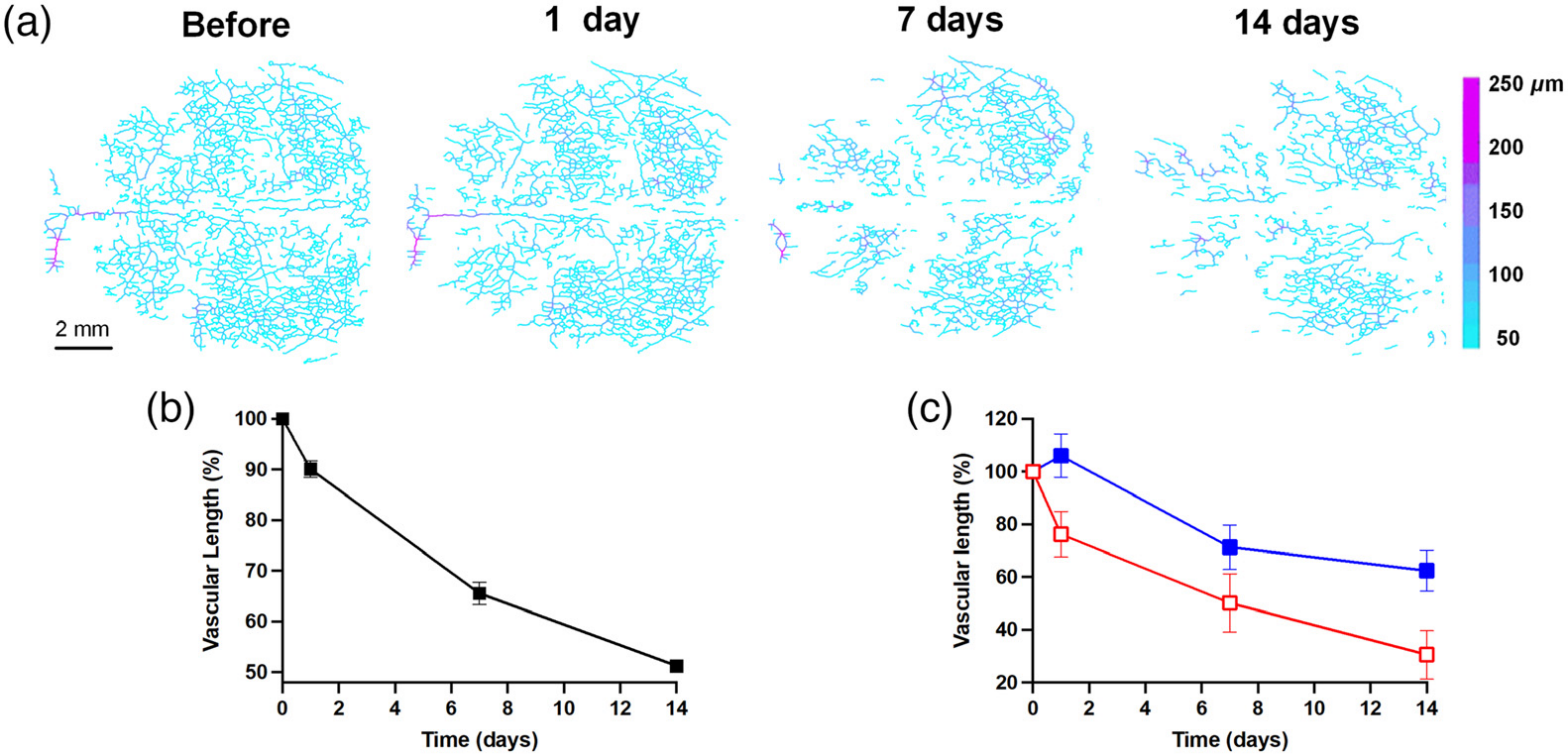
The progression of VL as a function of time after amyloid peptide perfusion in CSF. (a) The vascular skeleton diagrams with each pixel value in the figure represent the radius of the blood vessel (unit: μm). (b) The total VL% changes over time and (c) comparison of VL% between the anterior (red hollow square) and posterior (blue solid square) regions of mouse vasculature. Data (mean ± S.E.M.) were presented as percentage over the value obtained before peptide treatment and were plotted with monitoring time.

**Fig. 5:**
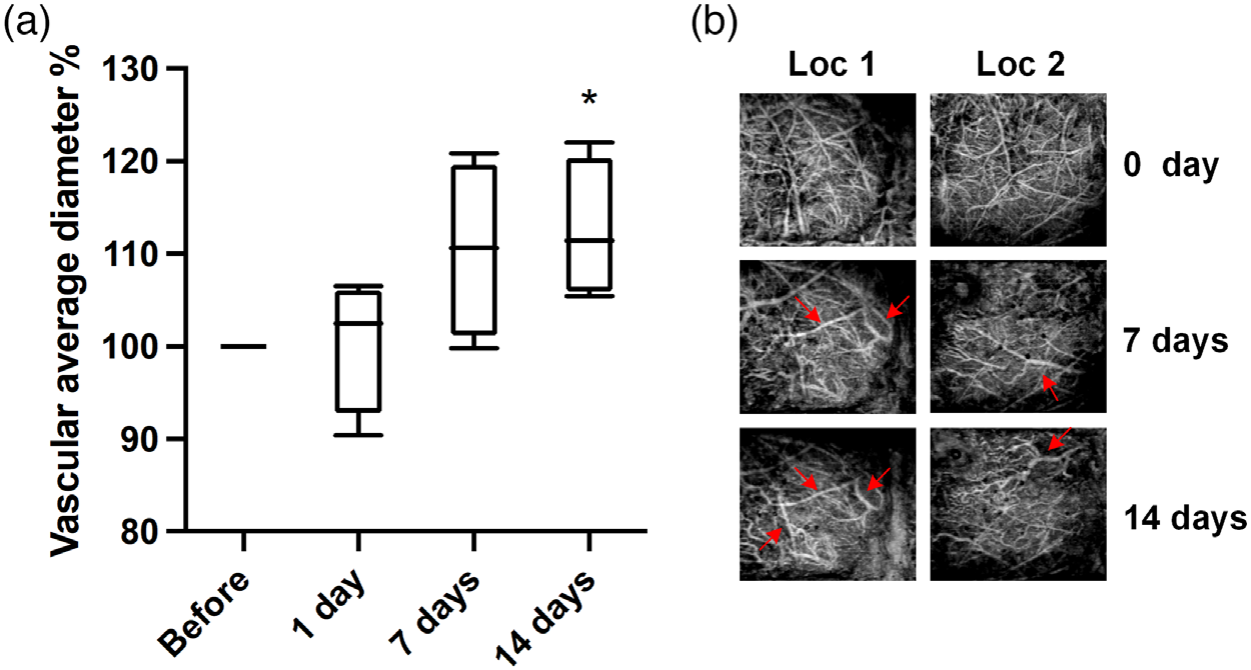
VAD changes after peptide injection. (a) Total VAD presented as a percentage of the value obtained before peptide treatment (before) and plotted over time. Each bar represents the group mean ± S.E.M, significant differences between certain group and before denoted *p<0.05. (b) Locations (Loc) where vascular diameters were visibly greater compared to untreated brain, indicated by red arrows. Each column represents the same location.

Next, we further investigated VL and VAD, the related parameters that may contribute to the decline of VPD. VL and VAD were calculated from the vascular skeleton images [Fig. 4(a)]. As shown in the results, VL decreased rapidly after peptide injection by ∼50% after 2 weeks [Fig. 4(b)]. Likewise, when comparing the front and back parts of the brain, the VL in the anterior part had a more drastic decrease than the posterior counterpart as shown in Fig. 4(c). In the case of VAD, there was a slight increase after 7 days post-injection, with the value reached a plateau of ∼111% of before treatment [Fig. 5(a)]. As can be seen from binarized angiographies, “vascular clusters” (Fig. 2 marked with dashed circles) began to form around the same time as the increase in VAD. This may contribute to the increase of total VAD value. In addition, some vessels appeared dilated [Fig. 5(b), red arrows], which can also lead to the slight enlargement of VAD value. On day 14, VAD values showed a significant difference (p=0.0452) compared with no treatment [Fig. 5(a)].

**Fig. 6:**
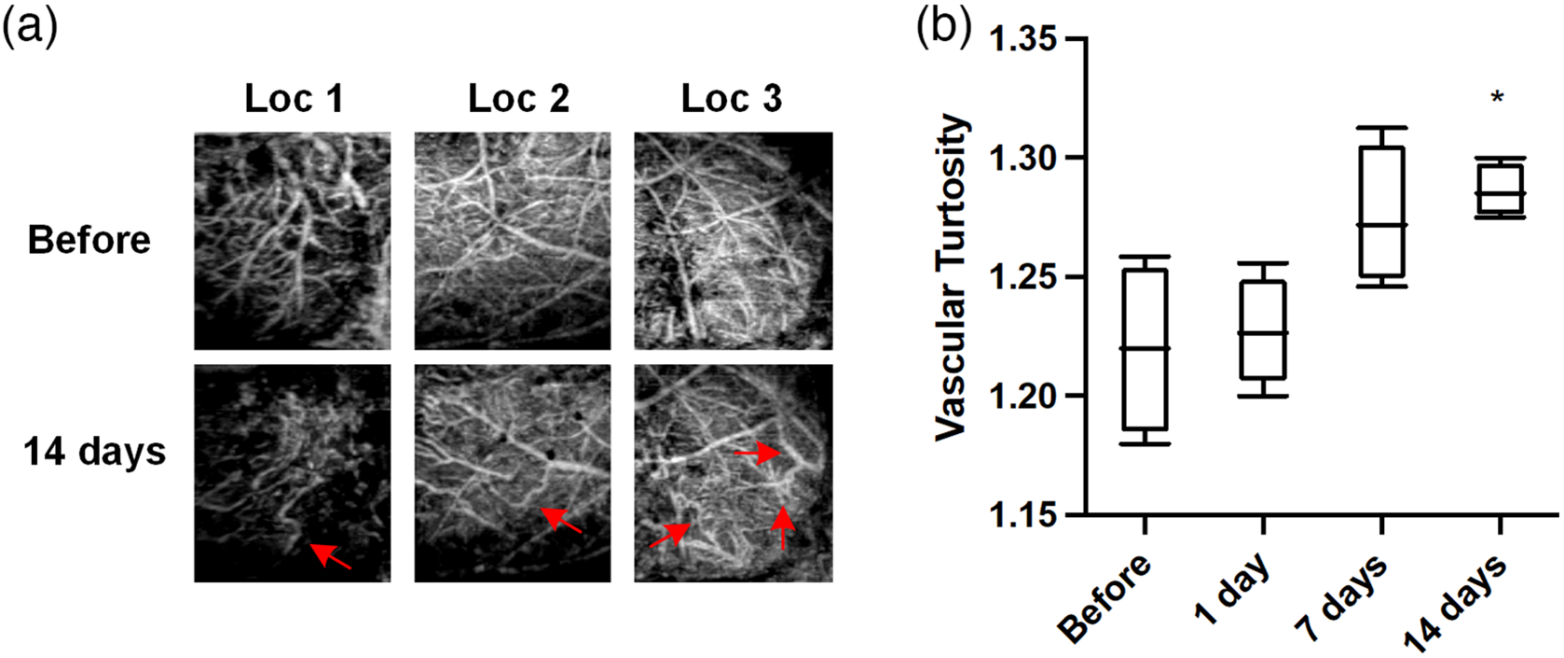
Cerebrovascular tortuosity before and after Aβ peptide exposure. (a) Regional OCT images showing that vessels became more tortuous at 14 days (second row, red arrows) compared to before treatment (first row). Pictures within the same columns are from the same location. (b) Average VT of the entire mouse brain. Each bar represents the group mean ± S.E.M, and significant differences between certain group and before peptide injection is denoted *p<0.05.

Distinct vascular morphological changes, such as tortuous, twisting, and kinking structures, are often observed during AD pathological progression, both in postmortem examinations and in AD transgenic mouse models. In this study, we also investigated the effect of CSF-Aβ on cerebrovascular curvature. Notably, some larger vessels appeared more tortuous 14 days after Aβ infusion [Fig. 6(a)]. The occurrence of tortuous and twisting [Fig. 6(a); Loc1 and 2] and kinking and looping [Fig. 6(a); Loc3] structures were observed. VT was quantified as the ratio of VL to the sum of Euclidean distances between the ends of each vascular branch within the entire mouse cerebrovasculature. As shown in Fig. 6(b), VT was modestly higher at 7 days (1.275±0.015, p=0.0521) and significantly greater at 14 days (1.286±0.006, p=0.0122) after peptide injection compared to without treatment (1.220±0.018).

**Fig. 7:**
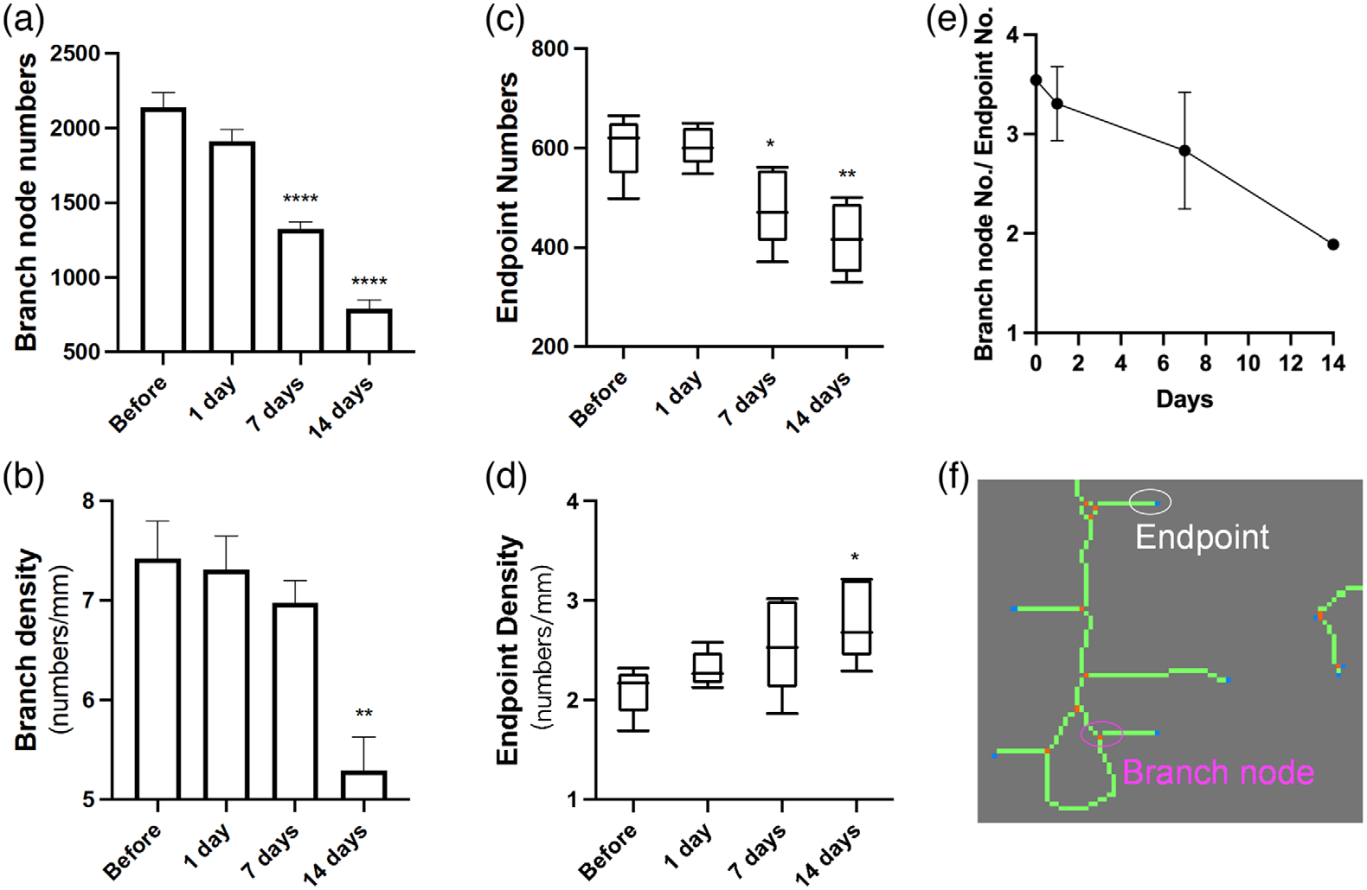
The effect of Aβ on vascular branching and fragmentation. (a) The branch node numbers based on calculating orange dots illustrated in (f) in the entire brain at different time points. (b) Branch density is defined as the branch node numbers divided by VL. (c) Endpoint numbers based on counting blue dots shown in (f) in the whole brain image. (d) Endpoint density is the ratio of endpoint numbers to the VL. (e) The ratio of branch node numbers/endpoint numbers is plotted over time. (f) A representative local area of the vascular skeleton image with branch nodes (orange dots) and endpoints (blue dots) labeled.

Ab influx in the CSF induced a continuous decrease in the total number of branch nodes [Fig. 7(a)], and when combined with changes in VL values over time, the branch density reduced significantly at 14 days [Fig. 7(b)]. This may indicate an anti-angiogenic effect. A significant decrease in numbers of endpoints was observed after 1 week [Fig. 7(c)]. This resulted in an increasing endpoint density as VL descended more rapidly throughout the monitoring time [Fig. 7(d)]. This could mean a rise in vascular fragments. In addition, the ratio of branch node numbers to endpoint numbers exhibited a steady decline with time [Fig. 7(e)], implying that the branch node numbers decreased faster than the endpoint numbers.

### 3.3 Aβ in CSF Reduces Global OAC of Mouse Brain

**Fig. 8:**
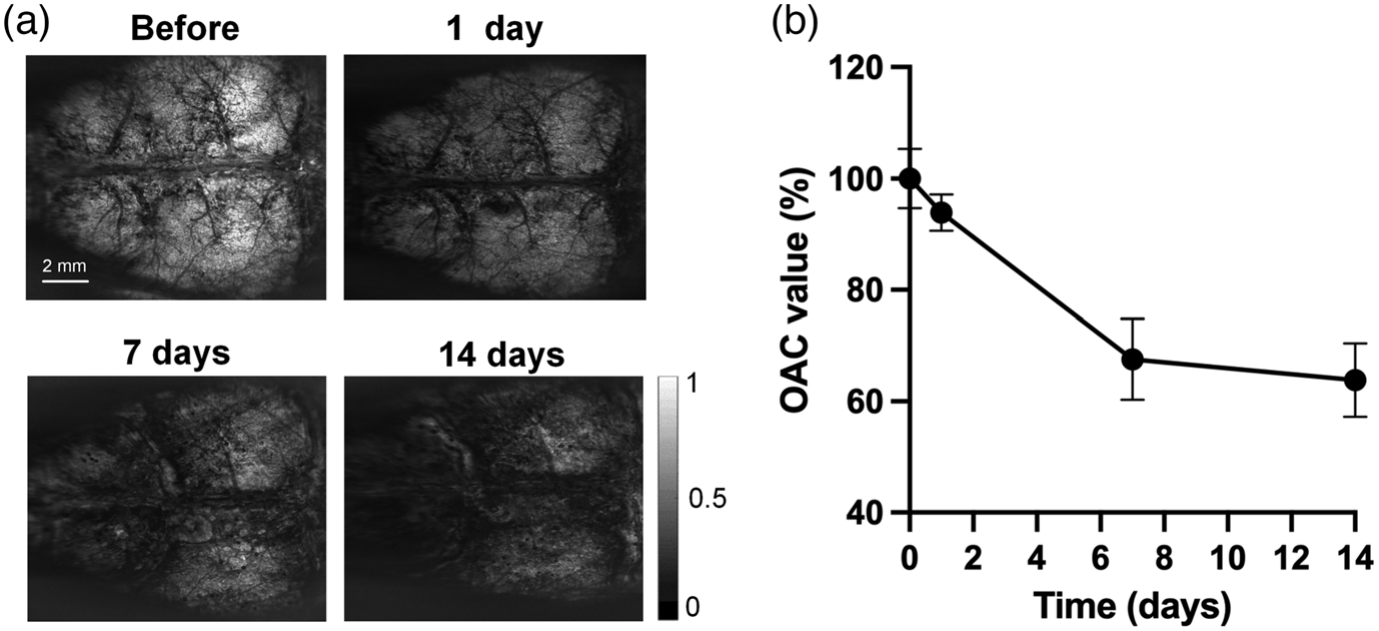
Aβ-induced changes in the average OAC of the brain over time. (a) The OAC images of the mouse brain at varies time points and (b) a quantification of the mean OAC value, expressed as a percentage at 0 day, presented as mean ± S.E.M.

OAC is an important parameter that measures the rate at which incident light is attenuated when passing through a medium, which enables quantitative analysis of tissue properties from OCT signals. In the study, we observed an overall reduction in OAC values initiating from the first day after peptide infusion [Figs. 8(a) and 8(b)]. This decline persisted with time, and after 2 weeks, the OAC in the mouse brain was reduced by 36.2% compared with the original level (day 0 value) [Fig. 8(b)]. This indicates systemic damage of the cerebral tissue.

## 4 Discussion

Continuous Aβ clearance through perivascular drainage ensures the amount of this protein maintains at a physiological level in the brain. Since the brain parenchyma is devoid of lymphatic vessels, glymphatic transport serves an active brain clearing function and is responsible for clearing neurotoxic metabolic wastes including Aβ and tau proteins.^23^^,^^48^ Glymphatic impairment underlines the pathological accumulation of Aβ in AD. Besides, ARIA diminishes the outcome of anti-Aβ immunotherapy, due to increased influx of soluble Aβ into the CSF that overwhelm the glymphatic system.^15^^,^^49^^–^^51^ However, the direct cerebrovascular consequences are not clear. As the two-hit hypothesis emphasizes the importance of brain vasculature in AD pathogenesis and supports the notion that understanding how blood vessels are altered is critical for developing future interventional strategies.^28^ We aimed to quantitatively analyze the progression of changes in cerebrovascular morphology and brain tissue properties through *in vivo* monitor of mouse cortex with $A\beta_{1-42}$ peptide overloaded in ventricular CSF using SS-OCT. This imaging modality allowed analysis of vascular networks throughout the entire mouse brain at relatively reasonable speed and resolution.

Our results showed that CSF $A\beta_{1-42}$ peptide-induced VPD reduction started from day 1 and continued to descend to 57.6% after 2 weeks. Many previous studies observed vascular density decline in AD mouse models and AD patient brain autopsies, particularly affecting small arterioles and capillaries in the hippocampus and cortex.^52^^–^^58^ The declined vascular density may be attributed to vascular occlusion resulted from accumulation of Aβ and fibrin, which lead to hypoperfusion.^53^^,^^59^ Moreover, perfusion deficit exacerbate and the resultant loss of endothelial cells may then account for the vascular density reduction at the stage.^10^ While several reports showed increased or no change in vascular density,^60^^,^^61^ it may be indicative of the remodeling of surviving vascular networks in AD brains. This idea is supported by a denser vascular networks around Aβ deposit in young APP23 mice.^62^ This increase in vascular density value may be temporary, attributed to transient release of vascular endothelial growth factor (VEGF).^53^^,^^58^^,^^63^ In contrast, a longitudinal study showed the transient rise was only observed in APP23 mice at 14 months of age, whereas a significant decrease in vascular density was found at 20 months of age, excluding aging factors.^53^ Nonetheless, our results aligned with observations in mice with advanced AD, and the diminished VPD value may result from both the reduction of perfusion, which might occur at earlier time points and the loss of vascular cells at later stage of monitoring. It should be noted that, from a methodological point of view, the parameter VPD of our study does not precisely correspond the vascular density suggested in above mentioned studies, especially *in vitro* postmortem examination that involves calculating stained endothelial cells as an index of vascular density.^54^ Whereas the parameter derived from OCT signals considered blood perfusion, and only functional vessels (vessels with blood flow) were counted. Nonetheless, our result suggests that soluble Aβ presence in CSF can also induce similar trends in VPD seen in AD patients and animal models.^52^^–^^58^ In addition, by analyzing regional VPD across the whole mouse brain, we also found that the frontal part of brain dropped more drastically than the posterior counterpart of this parameter, indicating the anterior part is a more vulnerable location for impairment onset and progression. The slight and transient increase in VPD and VL on day 1 [Fig. 3(d) and 4(c)] were consistent with previous reports on transgenic mice.^53^ The effect was considered compensatory, as the activation of macrophages and monocytes was activated, which caused the release of cytokines, such as VEGF, basic fibroblast growth factor, and platelet-derived growth factor.^53^ However, why the transitory rise in the parameters occurred in the posterior part rather than the anterior counterpart is not clear, nevertheless, it can be interesting to investigate in future studies.

In clinical trials of Aβ immunotherapy for AD, mounting evidence shows anti-Aβ monoclonal antibodies provoke significant increases in CSF Aβ levels,^15^^,^^49^^–^^51^ which may explain why these agents failed to meet their primary endpoints. VEGF, a potent stimulator of endothelial cell proliferation and angiogenesis, was found at heightened level in CSF of AD patients.^64^ When $A\beta_{1-42}$ was influxes into CSF, it competitively antagonizes VEGF binding to VEGF receptor-2 on endothelial cells,^65^ thereby reducing VEGF availability in the vasculature.^66^^,^^67^ This not only suppresses vessel sprouting, but more importantly, causes the regression of existing blood vessels, leaving behind “string vessel” structures—nonfunctional capillary remnants mostly composed of connective tissue and lacking in endothelial cells.^68^ This process may be relevant to AD pathology and may also explain the significant reduction in VL we observed during the 2-week monitoring period. Although further studies are required to confirm the occurrence of string vessels, our finding that $\mathrm{CSF}\text{-}A\beta_{1-42}$ promoted VL reduction agreed with previous observations in AD patient and mice, revealing Aβ-dependent vascular degeneration.^55^^,^^69^^–^^71^ Both VPD and VL in the cortex are sensitive to Aβ present in CSF and might be useful indicators for monitoring Aβ clearance via glymphatic transport pathway.

VAD is the averaged vascular diameter of the entire vasculature, which calculated from the OCT signal intensity along the vessel skeleton (central axis of vessel) after distance transformation from binarized OCT angiograms. In this study, a moderate but significant increase in VAD (∼111%) was observed 14 days after $A\beta_{1-42}$ CSF infusion, indicating a mild dilating effect of CSF amyloid. Hypoperfusion and hypoxia in AD mice can lead to vasoconstriction through upregulation of vascular contractile factor endothelin-1.^72^ In addition, Aβ *per se* is an endothelin-1 activator and can exhibit direct vessel contraction effects. This seems to contradict our results. However, this constriction may be transient.^73^ Interestingly, when AD transgenic mice (APP23) were monitored for 20 months, it showed an increase in mean vessel diameter and vessel size index, despite a slight constriction that occurred at 14 months.^53^ Our results align more with the pathological features at a late stage of AD. Similar to the patterns of decreased VPD and VL [Figs. 3(d) and 4(c)], morphological changes such as “bundles of vessels” were more pronounced in the frontal part of the mouse brain than its posterior counterpart (Fig. 2). Why the vessels in frontal portion of brain are more vulnerable to the toxic peptide is not clear. Zhang et al.^69^ proposed that cerebral areas with lower original vascular density are more susceptible to the deficiency in blood supply. This may explain our findings since we found that vascular density in the frontal brain was lower than that in the posterior counterpart in most of the mice studied.

Vascular deformations, such as tortuous, twisted, and looped vessels [Fig. 6(a)] and vascular bundles (Fig. 2; day 7 and 14) were observed in the later stage of our monitor. These vascular morphological alterations were also seen in many patients of advanced AD.^10^^,^^54^^,^^74^^,^^75^ Through calculating the ratio of actual distances and straight distances between ends of each branch, the labeled skeleton OCT images enabled us to quantify this vessel distortion morphology. Results revealed a moderate but significant increase in vascular curvature. These structural changes might exert significant impact on local blood flow, with abnormal structures contributing to increased vascular resistance and disturbing the overall hemodynamic state of the local vascular network.^54^^,^^76^ The increased density of string vessels as a result of endothelial degeneration is likely related to decreased local perfusion and metabolic dysfunction.^10^ Nonfunctional vessels might also come from aberrant angiogenesis, partly owning to the antagonizing effect of Aβ against VEGF.^65^ Our result supports this idea as we observed a significant reduction in branch density 14 days post-injection [Fig. 7(b)], indicating Aβ present in glymphatic system exerted anti-angiogenic and antibranching properties, in agreement with earlier report.^77^ Aβ prevents vessel sprouting, mainly through suppressing VEGF expression, secretion, and function,^65^^,^^78^^,^^79^ and supplement of VEGF in AD transgenic mice exhibits cognitive improvement, angiogenesis enhancement, and amyloid load reduction.^80^^,^^81^ Despite there is also evidence that Aβ can upregulate angiogenesis, these effects may be local and inefficient, with poor neovascularization and prone to premature regression due to vascular cell death and growth factor downregulation.^82^^,^^83^ We also measured endpoint density, calculated by the number of branch endpoints per mm of vessels. Although VL decreased over time [Fig. 4(b)], the endpoint density increased [Fig. 7(d)], indicating an increment of vessel fragments or a greater incidence of residual microvessels that are acellular and collapse, a microvascular morphology seen in advanced AD.^84^ It was also interesting to observe that the ratio of branch node numbers to endpoint numbers descended steadily over time, which might suggest a rich vascular network became sparser and more disconnected. Measurements of branch node density and endpoint density are very important parameters for the estimation of hypoperfusion and regional hypometabolism on a morphometric basis. Clearly, vascular structure and function influence and restrict each other.

OAC measures how quickly incident light decays when passing through a medium, enabling quantitative assessment tissue properties from OCT data. It is a reliable parameter for tissue differentiation and enhancing the diagnostic value of OCT. OAC has been used to detect atherosclerotic plaques,^85^ axillary lymph node imaging,^86^ to examine glaucoma,^87^ and to identify cancerous tissue from normal tissue in the bladder^88^ and colon.^89^ We have also successfully utilized it to assess the water content and cerebral edema resulted from cerebral infarction.^45^^,^^90^ In this study, the average OAC value of brain tissue decreased dramatically over time after ventricular injection of Aβ (Fig. 8). Aβ-induced vascular anomalies increases BBB permeability, causing extracellular accumulation of fluid and extravasation of serum proteins, which are the main features of vasogenic edema.^9^^,^^91^ Since the transmittance of infrared light from OCT system increases when water content of brain tissue is elevated, the value of OAC decreases accordingly. This makes OAC a competent parameter to distinguish edema tissue from normal tissue.^45^^,^^90^ We found that OAC value reduced uniformly throughout the brain, indicating a global edema condition, coincided with the vasogenic edema observed with MRI hyperintensities in ARIA.^12^^,^^92^

This study also has limitations. The changes in concentration of exogenous $A\beta_{1-42}$ peptide remained in glymphatic system are also a valuable parameter to examine. As our results suggested that soluble Aβ within glymphatic system is as toxic if not more toxic than the Aβ plaque and should not be neglected. Our further work will concern developing an Aβ tracer that allows real time *in vivo* detection by OCT. This could establish the dose-dependent relationship between CSF Aβ and brain vascular parameters.

In summary, this study reported the direct effects of CSF-Aβ on cerebrovascular morphology and brain tissue. The progression of changes in cortical microvascular morphology was examined *in vivo* by SS-OCT over a time course of 14 days. Remarkable and sustained decline in VPD, VL, branch density was determined, and slight but significant elevation in VT and VAD was observed 14 days post-injection. Endpoint density increased. Meanwhile, the global OAC reduction in the mouse brain also indicated the toxic effects of CSF-Aβ. The above results are consistent with the vascular pathological alterations often seen in advanced AD, which indicates a severe detrimental characteristic of $A\beta_{1-42}$ overloaded in glymphatic clearance system. Our work also demonstrates that OCT can be employed in drug discovery studies as a potential tool to monitor the brain vascular and tissue alterations in response to therapy.

## Code, Data, and Material Availability

All data used in this study are available from the first or corresponding author upon reasonable request and in accordance with institutional policies.
